# The draft genome of *Labeo catla*

**DOI:** 10.1186/s13104-020-05240-w

**Published:** 2020-09-03

**Authors:** Lakshman Sahoo, Paramananda Das, Bismay Sahoo, Gargee Das, Prem Kumar Meher, Uday Kumar Udit, Kanta Das Mahapatra, Jitendra Kumar Sundaray

**Affiliations:** Fish Genetics and Biotechnology Division, ICAR-Central Institute of Freshwater Aquaculture, Bhubaneswar, Odisha 751002 India

**Keywords:** *Labeo catla*, Hybrid assembly, Genomics resource, Indian major carp

## Abstract

**Objective:**

*Labeo catla* (catla), one of the three Indian major carps, is native to the Indo-Gangetic riverine system of India as well as the rivers of Pakistan, Bangladesh, Nepal and Myanmar. Its higher growth rate and compatibility with other major carps, specific surface feeding habit, and consumer preference have increased its popularity in carp polyculture systems among the fish farmers in Indian subcontinent. Recent advancement in sequencing technology coupled with massive parallel sequencing platforms has facilitated accelerated genetic improvement in aquaculture species through integration of genomics tools. A draft genome and allied resources are lacking in catla. Therefore, in the present study, we have performed *de-novo* assembly of *Labeo catla* for the first time.

**Data description:**

A male farm reared catla was used for extracting high molecular weight genomic DNA followed by sequencing in Oxford Nanopore and Illumina platforms. Approximately, 80× coverage of sequence data was assembled adopting the hybrid assembly strategy. The assembled genome size of catla was 1.01 Gb containing 5345 scaffolds with N50 value 0.7 Mb and more than 92% BUSCO completeness. Gene annotation resulted in 25,812 predicted genes.

## Objective

Aquaculture is the rapidly emerging food production sector all over the world and it is going to be the primary source of fish and shellfish for human diet in the coming future [1]. Genetic improvement of performance traits has huge potential to meet the increasing demand of quality animal protein in the event of exponential growth of human population. Well-designed breeding programmes integrated with genomics tools can accelerate the production and productivity. Recent advancement in sequencing technology coupled with massive parallel sequencing platforms has paved the way for expediting genetic improvement programs in aquaculture species.

*Labeo catla* (catla), one of the Indian major carps, is native to the Indo-Gangetic riverine system of India as well as the rivers of Pakistan, Bangladesh, Nepal and Myanmar. Its higher growth rate and compatibility with other major carps, specific surface feeding habit, and consumer preference have increased its popularity in carp polyculture systems among the fish farmers in India, Bangladesh, Myanmar, Laos, Pakistan and Thailand [2]. *L. catla* currently accounts for ∼ 3.4% of total freshwater aquaculture production worldwide [3]. With an aim to generate consolidated genomics resource for supporting genetic improvement, we have undertaken *de-novo* assembly of catla for the first time. The draft genome will also be an important resource for comparative genomics, biological and evolutionary studies of cyprinid species.

## Data description

One farm-reared mature (2 years old) male catla weighing approximately 1.7 kg was collected from ICAR-Central Institute of Freshwater Aquaculture (CIFA) farm for this study. Before tissue sampling, fish was anesthetized with MS-222 (300 mg/l) and then weighed. High molecular weight genomic DNA was isolated from testis tissue using standard phenol–chloroform method [4]. The qualitative and quantitative assessment of DNA were performed by NanoDrop spectrophotometer (Thermo Fisher Scientific, Wilmington, DE, U.S.A.) and Qubit fluorometer (Invitrogen, Carlsbad, CA, USA) followed by checking on 0.8% agarose gel. Genomic DNA was sheared using a Covaris S2 sonicator (Covaris, Woburn, Massachusetts, USA) to generate fragments in the range of 200 bp to 20 kb. Four Paired end libraries (insert size: 350 bp) for Illumina Nextseq500 platform and one library (mixed insert size) for Oxford Nanopore were prepared and sequenced as per manufacturer’s instruction. A total of 80.28 Gb sequence data (Table 1, Data file 1) [5] were generated after quality check by FastQC tool [6]. The de novo hybrid assembly was performed with default parameters using MaSuRCA 3.2.8 [7] followed by scaffolding and Gap closing with SSPACE v3.0 [8] and GapCloser v1.12b [9], respectively. This yielded 5,345 scaffolds with N50 value of 0.7 Mb (Table 1, Data file 2) [10] and largest fragment of 6.8 Mb. The assembled genome size of catla is 1.01 Gb (Table 1, Data file 3) [11] against an in silico estimated genome size of 0.95 Gb. The evaluation of genome by Benchmarking Universal Single-Copy Orthologs (BUSCO) version 3.0 [12] and using Actinopterygii odb9 core gene set revealed 92% complete, 87.9% complete and single copy, 4.1% complete and duplicated, 4.1% fragmented and 4.05% missing BUSCOs. RepeatModeler [13] was used for de novo repeat modelling which showed 47.58% of repeat content in catla genome. The genome wide simple sequence repeats of assembled catla genome was 391,331.

The catla genome is predicted to contain 25,812 protein-coding genes. Additionally, scaffold_2219 of a size of 16,600 bp, was found to be of mitochondrial origin, with 13 mRNAs, 22 tRNAs and 2 rRNAs. Functional annotation of the final set of predicted protein sequences was carried out by BLAST2GO v5.0. Out of 25,812 genes, 17,500 were found to have GO term assigned to them. The number of protein coding genes identified in catla (25,812) is comparable to the genomes of sequenced diploid cyprinids such as *Labeo rohita* [14]*, Ctenopharyngodon idellus* [15]*, Danio rerio* [16] and *Anabarilius grahami* [17]. Orthologous relationship among these species using OrthoVenn [18] showed a total of 8,494 orthologous gene clusters to be shared by all five species, with 1,357 species specific gene clusters. The whole genome sequence data has been deposited in the GenBank (Table 1, Data file 4) [19

**Table 1:** Overview of data files/data sets

Label	Name of data file/data set	File types (file extension)	Data repository and Identifier (DOI or Accession number)
Data file 1	Sequence data	Table 1.docx	https://doi.org/10.6084/m9.figshare.12271589 [5]
Data file 2	Assembly statistics	Table 2.docx	https://doi.org/10.6084/m9.figshare.12271619 [10]
Data file 3	Assembly data	FASTA	https://www.ncbi.nlm.nih.gov/assembly/GCA_012976165.1 [11]
Data file 4	Whole genome sequence data	FASTA	NCBI GenBank (Accession numbers VONZ01000001-VONZ01005345) https://identifiers.org/ncbi/insdc:VONZ00000000 [19]

## Limitations

The assembled genome size of *Labeo catla* is 1.01 Gb constituting 5345 scaffolds. The number of unassembled regions is 649 and the number of bases positioned in this gap is 0.8 Mb.

## Data Availability

This Whole Genome project has been deposited at DDBJ/ENA/GenBank under the Bioproject id: PRJNA557138 and Acc no: VONZ00000000 (https://identifiers.org/ncbi/insdc:VONZ00000000) [19]. The version described in this paper is version VONZ01000000. Please see Table 1 and references [5, 10, 11, 19] for details and links to the data.

## Abbreviations

FAO: Food and Agriculture Organisation of the United Nations
Gb: Giga base pair
MaSuRCA: Maryland Super-Read Celera Assembler
BUSCO: Benchmarking Universal Single-copy Orthologs
GO: Gene ontology
Mb: Mega base pair
